# Disease spread in age structured populations with maternal age effects

**DOI:** 10.1111/ele.12745

**Published:** 2017-03-07

**Authors:** Jessica Clark, Jennie S. Garbutt, Luke McNally, Tom J. Little

**Affiliations:** ^1^ Institute of Evolutionary Biology The University of Edinburgh Ashworth Laboratories Kings Buildings Charlotte Auerbach Road Edinburgh EH9 3FL Scotland; ^2^ Centre for Immunity Infection and Evolution The University of Edinburgh Ashworth Laboratories Kings Buildings Charlotte Auerbach Road Edinburgh EH9 3FL Scotland

**Keywords:** Age, *Daphnia*, demography, ecology, epidemiology, evolution, immunity, maternal effects, modelling, senescence

## Abstract

Fundamental ecological processes, such as extrinsic mortality, determine population age structure. This influences disease spread when individuals of different ages differ in susceptibility or when maternal age determines offspring susceptibility. We show that *Daphnia magna* offspring born to young mothers are more susceptible than those born to older mothers, and consider this alongside previous observations that susceptibility declines with age in this system. We used a susceptible‐infected compartmental model to investigate how age‐specific susceptibility and maternal age effects on offspring susceptibility interact with demographic factors affecting disease spread. Our results show a scenario where an increase in extrinsic mortality drives an increase in transmission potential. Thus, we identify a realistic context in which age effects and maternal effects produce conditions favouring disease transmission.

## Introduction

The ecological context of a host species will shape key demographic features, which in turn affect disease spread. Extrinsic mortality, for example, will regulate population density, a well‐established demographic feature that modulates disease spread. However, extrinsic mortality will shape other population characteristics such as age structure. The age structure of a population could affect disease dynamics through a number of mechanisms, including (1) when individuals of different ages show different susceptibilities (i.e. age effects) or (2) when the offspring of mothers of different ages show different susceptibilities (i.e. maternal age effects).

The age of a host at exposure to a parasite is well established to affect the probability and severity of infection, this is partly due to the ontogeny of the immune system (Lesser *et al*. 2006; Nussey *et al*. 2008; Hasselquist & Nilsson 2009). In vertebrates, this includes the development of adaptive immunity followed by immunosenescence, but invertebrates also appear to show a clear development of the immune system or pathogen resistance (Rheins & Karp 1985; Wilson‐Rich *et al*. 2008; Piñera *et al*. 2013; Garbutt *et al*. 2014a; Izhar & Ben‐Ami 2015; Izhar *et al*. 2015). The consideration of age‐related effects on epidemiological processes has contributed greatly to disease prevention strategies (Anderson & May 1982; Katzmann & Dietz 1984; Müller 2000).

By contrast, maternal age effects on offspring susceptibility to pathogens have received little attention. Generally, maternal effects have been shown to affect population dynamics and demography through alterations in offspring reproduction, maturation and growth rate (Gaillard *et al*. 2003; Benton *et al*. 2005; Beamonte‐Barrientos *et al*. 2010), and maternal effects have been shown to alter population robustness in the face of ecological challenges (Räsänen & Kruuk 2007; Kuijper & Hoyle 2015). The maternal condition, for example nutrition availability or disease status, is increasingly recognised to affect offspring susceptibility to infection (Huang & Song 1999; Little *et al*. 2003; Rahman *et al*. 2004; Mitchell & Read 2005; Gasparini *et al*. 2007; Lorenz & Koella 2011; Stjernman & Little 2011; Tidbury *et al*. 2011; Boots & Roberts 2012; Garbutt & Little 2017). This suggests variation in phenotypic traits, as a response to the maternal environment, can drive population and epidemiological dynamics (Beckerman *et al*. 2002; Mitchell & Read 2005; Garbutt *et al*. 2014b).

Maternal age has been shown to affect offspring performance as offspring of older mothers are often born larger and mature at a greater size (Priest *et al*. 2002; Benton *et al*. 2008). However, although older mothers produce larger offspring, they tend to produce fewer of these, suggesting they are subject to a ‘size vs. number’ trade‐off. These effects on performance measures indicate that maternal age effects may alter the competitive environment experienced by successive generations (Beckerman *et al*. 2006; Kindsvater *et al*. 2011; Prior *et al*. 2011).

To understand how maternal age influences susceptibility, we studied four clutches of offspring from the parthenogenic crustacean *Daphnia magna* when exposed to a bacterial pathogen. In addition to pathogen resistance, we collected data on reproduction and body size. The experiment showed that older mothers give birth to more resistant offspring. Earlier work from our laboratory (Garbutt *et al*. 2014a) and others (Izhar & Ben‐Ami 2015; Izhar *et al*. 2015) showed that older mothers themselves are more resistant to this pathogen. We used these two observations – that older mothers are both more resistant and give birth to offspring that are more resistant – to develop a compartmental model describing how population age structure, the effects of age and maternal effects combine to influence the spread of infection.

## Study organisms

*Daphnia magna* (Crustacea: Cladocera) are filter feeding planktonic crustaceans found in small freshwater ponds. *Pasteuria ramosa* is a sterilising specialist bacterial pathogen of *D. magna* that is transmitted horizontally when spores released from infected cadavers are ingested by uninfected filter feeding hosts (Ebert *et al*. 1996, 2016). In this experiment, we used a *Daphnia* clone (Kc49a) from a population in a pond in Kaimes in the Scottish borders, and a *P. ramosa* strain isolated from the same population. This host clone was part of a study of genetic variation in maternal effects (Stjernman & Little 2011) and shows a response that is typical of its population (see Little & Colegrave 2016 for discussion of the merits of single vs. multigenotype studies).

## Methods

The basic design of our experiment was to measure the pathogen susceptibility of offspring from first, second, third and fifth clutches. Two newborn *Daphnia* were isolated from each clutch. One of these offspring was exposed to *P. ramosa*. The second offspring was used for measurement of reproduction. Each newborn also had their body size measured before being placed into their treatment groups. This experiment ran from July 31–September 24 2014.

### Acclimation

Twenty‐four replicates, each an individual *Daphnia* in a 60 mL glass jar, were acclimatised for three generations under standardised conditions. This process is designed to equilibrate uncontrolled maternal effects and ensure that each replicate is independent. During this time, *Daphnia* were kept in artificial pond medium (Kluttgen *et al*. 1994) in an incubator with a light:dark cycle of 12 : 12 L:D at 20 °C. They were fed daily with 7 × 10^6^ cells of green algae, *Chlorella* spp., and changed into fresh medium twice a week and when offspring were present in the jar. Each new generation was initiated with offspring from the second clutch. After the third homogenising generation, one individual was taken from each replicate and placed in fresh jars to be the mothers of the experimental animals. These individuals were kept in the same conditions as the homogenising generations.

### Experimental generation

#### Experiment 1

Offspring from clutch 1, 2, 3 and 5 of the maternal generation were our experimental animals. Two offspring were taken from each mother and were randomly assigned to either pathogen exposure or control – used for the measurement of fecundity in the absence of infection. For pathogen exposure, 20 000 *P. ramosa* spores were added to each jar and media was not changed for 5 days. Spore mixes were prepared earlier by crushing infected *Daphnia* and counting spores with a haemocytometer. At the end of the 5‐day exposure treatment, *Daphnia* were changed into clean jars with fresh media. *Daphnia* were maintained for 28 days after the exposure period under the conditions used for acclimation. During this period, the date of birth of each clutch and number of individuals born to each clutch was recorded. At the end of the 28‐day period, infections were diagnosed (infections are easy to discern with the naked eye as *Daphnia* have a clear carapace and reddish‐brown bacterial growth is visible in the haemolymph in addition to the lack of reproduction). The control *Daphnia* whose fecundity was measured were handled identically, except they were not exposed to parasite spores. All newborn *Daphnia* were photographed for later measurement of size at birth.

#### Independent replication of experiment 1

The design and methodology of this experiment were identical to that detailed above: we measured the fitness of offspring from first, second, third and fifth clutches following identical protocol for pathogen exposure and control individuals.

#### Analysis

Body size at birth and total reproduction were analysed in a general linear model. Age at first reproduction was studied with a parametric survival analysis following a Weibull distribution. A generalised linear model (with binomial errors and logit link function) was used to study probability of becoming infected. In all cases, the explanatory variable was the clutch the *Daphnia* originated from. The different response variables were analysed with different subsets of the data: size at birth considered the entire data set, reproduction considered only *Daphnia* that were unexposed to *P. ramosa*, while the probability of infection included only *Daphnia* that were exposed to the pathogen.

## Experimental Results

**Figure 1:**
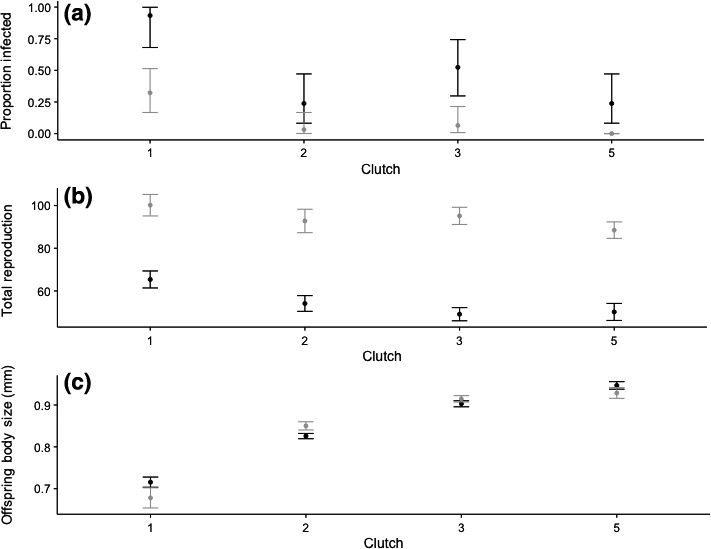
Both the original experiment (shown in black) and independent replication of the experiment (shown in grey) showing (a), proportion of infections resulting from exposure of the treatments groups from clutches 1, 2, 3 and 5, (b) reproductive output and (c) the effect of clutch number (maternal age at reproduction) on offspring body size.

The age of the mother had a significant effect on the probability of offspring infection (*χ* ^2^ = 24.8, *P* < 0.0001) where offspring from young mothers were highly susceptible to infection, as shown in Fig. 1a. Maternal age (or variable ‘Clutch’ for clutch number) also had a significant effect on total reproduction (*F*_3,72_ = 15.8, *P* < 0.0001) as seen in Fig. 1b and size at birth as seen in Fig. 1c (*F*_3,91_ = 219, *P* < 0.0001). Age at first reproduction was not influenced by maternal age (*χ* ^2^ = 5.07, *P* = 0.16). These patterns were confirmed through the independent replication of the experiment. Maternal age had a significant effect on the probability of becoming infected (*χ* ^2^ = 20.6, *P* < 0.0001; Fig. 1a), total reproduction (*F*_3,76_ = 3.91, *P* < 0.002; Fig. 1b) and size at birth (*F*_3,105_ = 96, *P *<* *0.0001; Fig. 1c).

## Epidemiological Model

Our experimental results showed a strong effect of maternal age on offspring resistance, with older mothers producing more resistant offspring. Previous research showed that *D. magna* show age‐specific susceptibility, where older individuals are more resistant to infection than young individuals (Garbutt *et al*. 2014a; Izhar & Ben‐Ami 2015; Izhar *et al*. 2015). Host population demography and environmental factors, such as baseline mortality rates, predation and density, have been shown to influence transmission rates and pathogen virulence evolution. Pathogen transmission is a function of an infected individual's rate of contact with other potential hosts. As increased host population density should increase contact rates, it is generally expected that an increase in population density will result in an increase in transmission potential (Anderson & May 1979; Ebert & Mangin 1997; Choo *et al*. 2003). The theoretical consideration of age‐specific effects on epidemiological dynamics tends to consider age‐specific mortality and age‐specific contact rates rather than the strength or weakness of an age‐associated immune response (Anderson & May 1982; Katzmann & Dietz 1984; Castillo‐Chavez *et al*. 1989; Müller 2000). In addition to this, the presence of maternal age effects on offspring susceptibility has not been considered. We therefore developed a compartmental model incorporating these age effects on the expected number of secondary infections resulting from the introduction of an individual infected with a novel pathogen into a completely susceptible population ($R_{0}$). *R*_0_ depends on the duration of infection, the probability of infecting a susceptible during one contact and the rate of contact per unit of time (Dietz 1993). The output value can be used as a benchmark. If the value of this is > 1, then the disease will spread (Anderson & May 1979).

**Figure 2:**
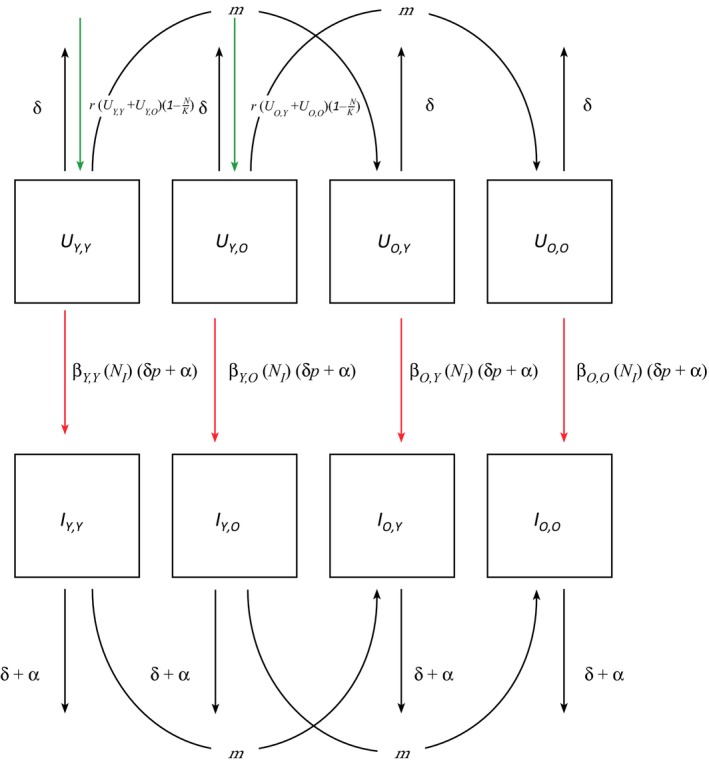
The compartmental model with uninfected and infected groups of four age classes. UY;Y – young individuals with a young mother. UY;O – young individuals with an old mother. UO;Y– old individuals with a young mother. UO;O – old individuals with an old mother. m is the rate at which an individual goes from being a young individual to an old individual. b is the rate of transmission. d is the baseline death rate, with d +a representing baseline death rate plus a measure of virulence. Other parameters are r – maximum reproduction; K – carrying capacity; N – total population number; *p* ‐ the probability that non‐pathogen induced death of an infected individual will lead to transmission. As *p*. ramosa is only transmitted upon host death, death rates are included in the transmission terms.

We divide the population into four age classes, each of which could be infected (*I*) or uninfected (*U*), giving a total of eight classes of individuals in the model – *U*_*Y,Y*_, *U*_*Y,O*_, *U*_*O,Y*_, *U*_*O,O*_, *I*_*Y,Y*_, *I*_*Y,O*_, *I*_*O,Y*_, *I*_*O,O*_ (Fig. 2). Here, the first subscript indicates the individual's age (*Y* for young, *O* for old), and the second subscript indicates the individual's mother's age at that individual's birth.

The parameters in the model are: β is the baseline transmission rate between infected and uninfected individuals; δ is the baseline death rate; α is pathogen virulence as measured by disease induced death; *p* is the probability that non‐pathogen induced death of an infected individual will also lead to transmission; *m* is the rate of maturation (i.e. the rate at which individuals move from the young age classes to the old); *M* describes the proportional reduction in an individual's susceptibility as associated by having a mother of the age class ‘Old’ applied to the baseline β; *A* describes the proportional reduction to an individual's susceptibility by themselves being of the age class ‘Old’ applied to the baseline β; *r* is the maximum per capita growth rate of hosts; *K* is the carrying capacity of the host population, controlling density dependent limitation on reproduction. The transmission terms, as shown in Fig. 2, contain the rates of death, due to *P. ramosa* being transmitted only upon host death. We make the assumptions that there is a constant rate of death across age groups that susceptibility varies between these classes, and that reproduction is resource limited with a constant maximum reproductive rate per day for uninfected individuals. We assume that infected individuals do not reproduce as *P. ramosa* castrates the host during infection (Ebert *et al*. 1996). For full details of the model, see the appendix.

In the absence of the pathogen, the equilibrium densities of each age class are

(1a) $$U_{Y,Y}=\frac{\left(r\delta^{2}-\delta^{3}\right)K}{r{\left(\delta +m\right)}^{2}}$$

(1b) $$U_{Y,O}=\frac{\delta m\left(r-\delta \right)K}{r{\left(\delta +m\right)}^{2}}$$

(1c) $$U_{O,Y}=\frac{\delta m\left(r-\delta \right)K}{r{\left(\delta +m\right)}^{2}}$$

(1d) $$U_{O,O}=\frac{m^{2}\left(r-\delta \right)K}{r{\left(\delta +m\right)}^{2}}$$

**Figure 3:**
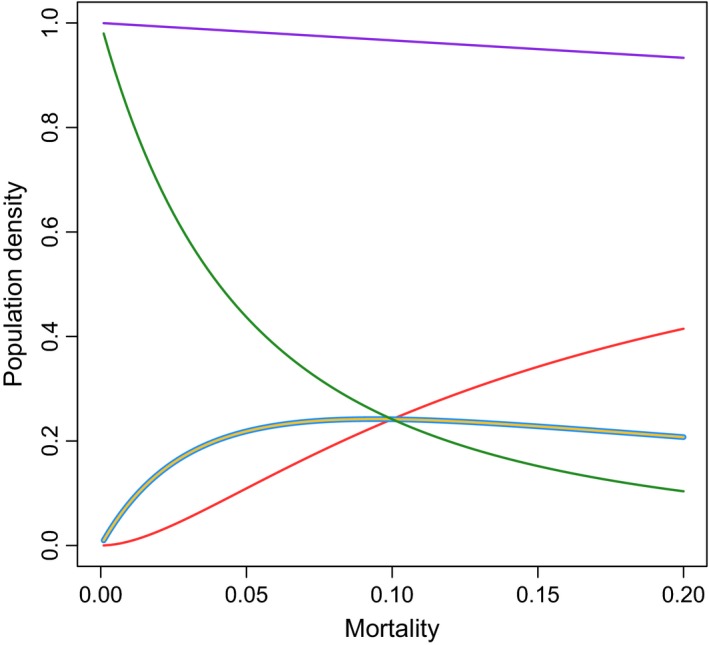
Showing the changes in total population density with increased mortality (purple line). This is broken down into the age classes showing density of young individuals with young mothers (red line), young individuals with old mothers (yellow line), old individuals with young mothers (blue line) and old individuals with old mothers (green line). Due to parameterization of the model, the yellow and blue lines trace one another. Other parameters are *r *=* *3; *K* = 1; *m *=* *1/10.

Here, we see that the equilibrium density of *U*_*O*,*O*_ decrease with increasing mortality δ (as *r *> δ is necessary for a positive equilibrium). The equilibrium density of both *U*_*Y*,*O*_ and *U*_*O*,*Y*_ initially increases with increasing mortality before decreasing once δ>2r+m. Similarly, the density of the age class *U*_*Y*,*Y*_ initially increases with increasing mortality before decreasing once $\delta >\left(\sqrt{m\left(9m+8r\right)}-3m\right)/2$ (Fig. 3). This bias of the age structure towards more susceptible younger individuals and individuals from younger mothers occurs as increased mortality frees up resources for reproduction, resulting in the production of more young individuals who will themselves reproduce leading to more individuals from young mothers. At this equilibrium, the total density of uninfected hosts, *N*_*U*_, is

(2) $$N_{U}=K\left(1-\frac{\delta }{r}\right)$$

meaning that the total density of uninfected individuals monotonically declines with increasing mortality rate (Fig. 3).

To calculate the *R*_0_ of the pathogen, we consider the number of secondary infections caused by a rare pathogen at the pathogen‐free equilibrium given in eqns 1a–d. This yields a pathogen *R*_0_ of

(3) $$R_{0}=\frac{\beta \left(r-\delta \right)\left(\alpha +\delta p\right)\left(\delta +m\left(1-M\right)\right)\left(\delta +m\left(1-A\right)\right)K}{r\left(\alpha +\delta \right){\left(\delta +m\right)}^{2}}$$

**Figure 4:**
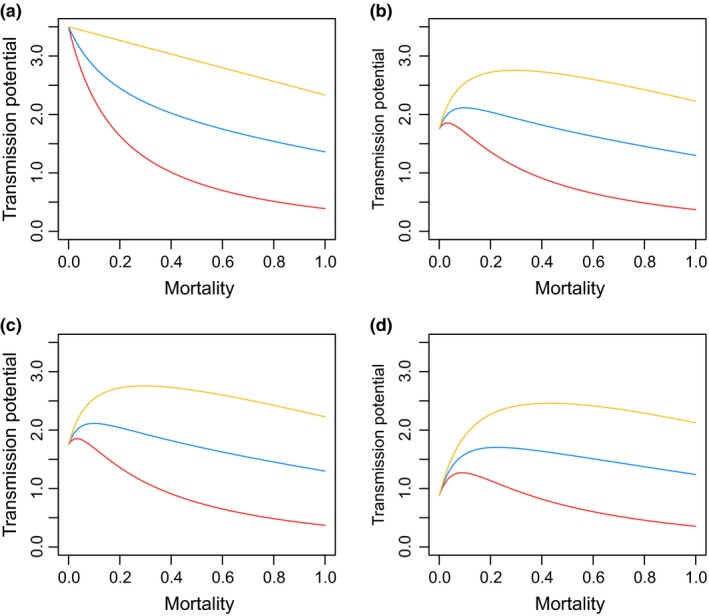
Modelling outputs of the relationship between mortality and transmission potential ($R_{0})$ with three different parameter values for *p* (extrinsic mortality of infected individuals contributing to transmission). *p* =* *1 (yellow line), *p* =* *0.5 (blue line) and *p* =* *0 (red line). (a) No age effects or maternal age effects present, (b) Maternal age effects, (c) Age specific susceptibility, or (d) – Both maternal and age effects present. In the presence of no effects (a), the expected negative relationship between mortality and transmission potential is shown at all levels of extrinsic mortality. The presence of maternal age effects (b), age‐specific susceptibility (c) or both effects (d) within a population results in a humped relationship, where an increase in mortality initially increases transmission, due to the shift in density of susceptible individuals. This positive relationship between mortality and transmission potential is most pronounced at all levels of extrinsic mortality when both effects are present. Other parameters are: *r *=* *3; *K *=* *1; *a *=* *1/5; *m *=* *1/10; $\beta_{Y,Y}$ **= 3.5.

In the appendix, we show that the relationship between pathogen *R*_0_ and the extrinsic death rate, δ, must be negative whenever our age‐related and maternal age‐related effects on susceptibility are absent (i.e. *A *=* *0, *M *=* *0). However, this relationship is humped whenever

(4) $$m<\frac{\alpha r\left(A+M-2AM\right)}{\left(1-A\right)\left(1-M\right)\left(\alpha +r\left(1-p\right)\right)}$$

with this condition satisfied for sufficiently large reductions in susceptibility with age and maternal age (high *A* and *M*). The relationship is illustrated with and without both the age‐ and maternal age‐related reductions in susceptibility in Fig. 4. These qualitative results also hold when an environmental transmission stage of the pathogen is explicitly included in the model (see Appendix).

The humped shape can be explained as follows. In the absence of reductions in susceptibility with age and maternal age, reductions in host population density with increasing mortality reduce pathogen *R*_0_ owing to reduced transmission. However, increased mortality also shifts the age structure of the population towards younger individuals and individuals from younger mothers, which can increase in density even as the total population density declines. If these age classes of individual are sufficiently more susceptible to infection, this increase in their density can more than compensate for the total reduction in host population density, leading to an increase in *R*_0_ with increasing non‐pathogen induced mortality. However, at very high mortality rates the total host population density is sufficiently reduced that shifts in age structure no longer compensate, and *R*_0_ declines with mortality, resulting in a humped relationship. Transmission potential begins to decline at lower mortality rates when, *p* (the probability that non‐pathogen induced death of an infected individual will lead to transmission) is low, as increasing mortality will result in loss of infections with little opportunity for transmission. Note, however that our qualitative results hold even in the case of *p* =* *0.

## Discussion

Our experiment makes the novel observation that younger mothers give birth to offspring that are more pathogen susceptible. This data combined with recent observations that younger hosts are themselves more susceptible (Garbutt *et al*. 2014a; Izhar & Ben‐Ami 2015; Izhar *et al*. 2015) formed the basis of a compartmental model showing that increasing extrinsic mortality, and thus decreasing total host population density, can lead to increasing transmission. The relationship between mortality and transmission that is classically seen, where *higher* host densities generate greater transmission, is so pervasive it has become a central assumption of the epidemiological theory that contributes to our understanding of pathogen traits and virulence. (Anderson & May 1979; Read 1994; Levin 1996; Alizon *et al*. 2009; Schmid‐Hempel 2011). Thus, we have identified a new, and biologically realistic context in which age effects and maternal effects combine to alter the ecological conditions favouring disease transmission.

Our observed effect of maternal age on offspring performance is an example of maternal effect‐driven, delayed life history effects (Beckerman *et al*. 2002) that lead to whole cohort effects (Ginzburg *et al*. 1994; Lindström 1999). The maternal condition has previously been shown to have prolonged effects on offspring fecundity, maturation and growth rate over successive generations, influencing population dynamics (Gaillard *et al*. 2003; Benton *et al*. 2005; Beamonte‐Barrientos *et al*. 2010). We now add offspring susceptibility to this list, and can infer from our model that maternal age effects on susceptibility can then impact successive cohorts, by altering the condition of each cohort at reproduction. Considering *P. ramosa* specifically, the predictions of our model are biologically feasible. This pathogen sterilises hosts, but often one clutch is produced before reproduction stops. If no effect of maturation or maternal effects were present, the transmission potential would decrease as seen in Fig. 4. In the presence of these effects, however, those who are young when infected only reproduce once whilst young and then do not contribute to the population at a later age. Their offspring are therefore expected to be the most susceptible. As mortality increases, the density of young individuals contributing highly susceptible individuals to the population increases, resulting in the dynamic we show.

The maternal effect of increased resistance in offspring born to older mothers could be explained by size at birth. Older mothers are known to produce larger offspring (Marshall *et al*. 2010; Kindsvater *et al*. 2011) and therefore the size at birth could be responsible to some degree for the resistance to disease. This correlation of body size to susceptibility has been made before in *Daphnia*, and it has been suggested this is due to increased resources availability for costly immune defences and somatic maintenance (Garbutt *et al*. 2014a). In *Daphnia spp*. and other systems, however, this correlation between increased size and reduced infection probability does not always hold true (Hall *et al*. 2007; Stjernman & Little 2011). Furthermore, the assumption that increased body size equates to better fitness and performance does not explain how smaller offspring of young mothers are capable of reproducing in higher numbers than their larger counterparts.

The observed trade‐off between offspring size and the decreasing number of offspring in each successive reproductive event could be adaptive if clutches of older mothers were to experience more challenging environments than young mothers. This is plausible given population size could increase, increasing competition, and potentially pathogen prevalence. It has been seen previously that *Daphnia* produce larger and more resilient offspring in tough conditions (Mitchell & Read 2005; Garbutt *et al*. 2014b) and larger individuals are better competitors for resources in a more dense populations (Brockelman 1975). In addition, individuals born to older mothers may need to be larger in order to compete with their older siblings (Plaistow *et al*. 2007).

In summary, our experimental results add evidence to a growing body of work showing maternal age effects on offspring performance (Berkeley *et al*. 2004; Plaistow *et al*. 2007; Marshall *et al*. 2010). Furthermore, our model shows that where age‐related and maternal age‐related effects occur they can fundamentally transform the ecological conditions that favour disease transmission. Divergent host susceptibilities within a population could also have marked effects on pathogen evolution and virulence, and our model lends itself to an extension that includes studies of optimal virulence.

## Statement of Authorship

JC wrote manuscript; JC and LM created model; TL and LM provided comments on the manuscript; JG and TL designed experiment; JG carried out laboratory work.

## Data Accessibility

Data available from http://doi.org/10.5281/zenodo.322882

## References

[ele12745-bib-0001] Alizon, S., A., H. , Mideo, N. & Van Baalen, M. (2009). Virulence evolution and the trade‐off hypothesis: history, current state of affairs and the future. J. Evol. Biol., 22, 245–259. 19196383 10.1111/j.1420-9101.2008.01658.x

[ele12745-bib-0002] Anderson, R.M. & May, R.M. (1979). Population biology of infectious diseases: Part I. Nature, 280, 361–367. 460412 10.1038/280361a0

[ele12745-bib-0003] Anderson, R. & May, R.M. (1982). Directly transmitted infectious diseases: control by vaccination. Science (80‐.)., 215, 1053–1060. 7063839 10.1126/science.7063839

[ele12745-bib-0004] Beamonte‐Barrientos, R. , Velando, A. , Drummond, H. & Torres, R. (2010). Senescence of maternal effects: aging influences egg quality and rearing capacities of a long‐lived bird. Am. Nat., 175, 469–480. 20175680 10.1086/650726

[ele12745-bib-0005] Beckerman, A. , Benton, T.G. , Ranta, E. , Kaitala, V. & Lundberg, P. (2002). Population dynamic consequences of delayed life‐history effects. Trends Ecol. Evol., 17, 263–269.

[ele12745-bib-0006] Beckerman, A.P. , Benton, T.G. , Lapsley, C.T. & Koesters, N. (2006). How effective are maternal effects at having effects? Proc. R. Soc. B Biol. Sci., 273, 485–493. 10.1098/rspb.2005.3315 PMC1560202 16615217

[ele12745-bib-0007] Benton, T.G. , Plaistow, S.J. , Beckerman, A.P. , Lapsley, C.T. & Littlejohns, S. (2005). Changes in maternal investment in eggs can affect population dynamics. Proc. Biol. Sci., 272, 1351–1356. 16006330 10.1098/rspb.2005.3081 PMC1560333

[ele12745-bib-0008] Benton, T.G. , St Clair, J.J.H. & Plaistow, S.J. (2008). Maternal effects mediated by maternal age: From life histories to population dynamics. J. Anim. Ecol., 77, 1038–1046. 18631260 10.1111/j.1365-2656.2008.01434.x

[ele12745-bib-0009] Berkeley, S.A. , Chapman, C. & Sogard, S.M. (2004). Maternal age as a determinant of larval growth and survival in a marine fish, Sebastes melanops. Ecolo, 85, 1258–1264.

[ele12745-bib-0010] Boots, M. & Roberts, K.E. (2012). Maternal effects in disease resistance: poor maternal environment increases offspring resistance to an insect virus. Proc. R. Soc. B Biol. Sci., 279, 4009–4014. 10.1098/rspb.2012.1073 PMC3427573 22833270

[ele12745-bib-0011] Brockelman, W.Y. (1975). Competition, the fitness of offspring, and optimal clutch size. Am. Nat., 109, 677–699.

[ele12745-bib-0012] Castillo‐Chavez, C. , Hethcote, H.W. , Andreasen, V. , Levin, S.A. & Liu, W.M. (1989). Epidemiological models with age structure, proportionate mixing, and cross‐immunity. J. Math. Biol., 27, 233–258. 2746140 10.1007/BF00275810

[ele12745-bib-0013] Choo, K. , Williams, P.D. & Day, T. (2003). Host mortality, predation and the evolution of parasite virulence. Ecol. Lett., 6, 310–315.

[ele12745-bib-0014] Dietz, K. (1993). The estimation of the basic reproduction number for infectious diseases. Stat. Methods Med. Res., 2, 23–41. 8261248 10.1177/096228029300200103

[ele12745-bib-0015] Ebert, D. & Mangin, K.L. (1997). The influence of host demography on the evolution of virulence of a microsporidian gut parasite. Evolution, 51, 1828–1837. 28565099 10.1111/j.1558-5646.1997.tb05106.x

[ele12745-bib-0016] Ebert, D. , Rainey, P. , Embley, M.T. & Scholz, D. (1996). Development, life cycle, ultrastructure and phylogenetic position of Pasteuria ramosa Metchnikoff 1888: rediscovery of an obligate endoparasite of Daphnia magna Straus. Philos. Trans. B, 35, 1689–1701.

[ele12745-bib-0017] Ebert, D. , Duneau, D. , Hall, M.D. , Luijckx, P. , Andras, J.P. , Du Pasquier, L. *et al* (2016). A Population Biology Perspective on the Stepwise Infection Process of the Bacterial Pathogen Pasteuria ramosa in Daphnia In: Advances in Parasitology (ed David Rollinson & Russell Stothard). Academic Press, Cambridge, MA, pp. 265–310. 10.1016/bs.apar.2015.10.001 27015951

[ele12745-bib-0018] Gaillard, J.M. , Loison, A. , Toigo, C. , Delorme, D. & Van Laere, G. (2003). Cohort effects and deer population dynamics. Ecoscience, 10, 412–420.

[ele12745-bib-0019] Garbutt, J.S. & Little, T.J. (2017). Bigger is better: changes in body size explain a maternal effect of food on offspring disease resistance. Ecol Evol., doi:10.1002/ece3.2709. PMC5330872 28261452

[ele12745-bib-0020] Garbutt, J.S. , O'Donoghue, A.J.P. , McTaggart, S.J. , Wilson, P.J. & Little, T.J. (2014a). The development of pathogen resistance in Daphnia magna: implications for disease spread in age‐structured populations. J. Exp. Biol., 217, 3929–3934. 25214486 10.1242/jeb.111260 PMC4213179

[ele12745-bib-0021] Garbutt, J.S. , Scholefield, J.A. , Vale, P.F. & Little, T.J. (2014b). Elevated maternal temperature enhances offspring disease resistance in Daphnia magna. Funct. Ecol., 28, 424–431.

[ele12745-bib-0022] Gasparini, J. , Boulinier, T. , Gill, V.A. , Gil, D. , Hatch, S.A. & Roulin, A. (2007). Food availability affects the maternal transfer of androgens and antibodies into eggs of a colonial seabird. J. Evol. Biol., 20, 874–880. 17465898 10.1111/j.1420-9101.2007.01315.x

[ele12745-bib-0023] Ginzburg, L.R. , Taneyhill, D.E. , Ginzburg, L.E.V.R. & Taneyhill, D.E. (1994). Population cycles of forest Lepidoptera : a maternal effect hypothesis. J. Anim. Ecol., 63, 79–92.

[ele12745-bib-0024] Hall, S.R. , Sivars‐Becker, L. , Becker, C. , Duffy, M.A. , Tessier Alan, J. & Caceres, C.E. (2007). Eating yourself sick : transmission of disease as a function of foraging ecology. Ecol. Lett., 10, 207–218. 17305804 10.1111/j.1461-0248.2007.01011.x

[ele12745-bib-0025] Hasselquist, D. & Nilsson, J.‐A. (2009). Maternal transfer of antibodies in vertebrates: trans‐generational effects on offspring immunity. Philos. Trans. R. Soc. B‐Biological Sci., 364, 51–60. 10.1098/rstb.2008.0137 PMC2666691 18926976

[ele12745-bib-0026] Huang, C.C. & Song, Y.L. (1999). Maternal transmission of immunity to white spot syndrome associated virus (WSSV) in shrimp (Penaeus monodon). Dev. Comp. Immunol., 23, 545–552. 10579383 10.1016/s0145-305x(99)00038-5

[ele12745-bib-0027] Izhar, R. & Ben‐Ami, F. (2015). Host age modulates parasite infectivity, virulence and reproduction. J. Anim. Ecol., 84, 1018–1028. 25661269 10.1111/1365-2656.12352

[ele12745-bib-0028] Izhar, R. , Routtu, J. & Ben‐ami, F. (2015). Host age modulates within‐host parasite competition. Biol. Lett., 11, 20–23. 10.1098/rsbl.2015.0131 PMC4455738 25994010

[ele12745-bib-0029] Katzmann, W. & Dietz, K. (1984). Evaluation of age‐specific vaccination strategies. Theor. Popul. Biol., 25, 125–137. 6729750 10.1016/0040-5809(84)90016-9

[ele12745-bib-0030] Kindsvater, H.K. , Bonsall, M.B. & Alonzo, S.H. (2011). Survival costs of reproduction predict age‐dependent variation in maternal investment. J. Evol. Biol., 24, 2230–2240. 21745251 10.1111/j.1420-9101.2011.02351.x

[ele12745-bib-0031] Kluttgen, B. , Dulmer, U. , Engels, M. & Ratte, H.T. (1994). ADaM, an artificial freshwater for the culture of zooplankton. Water Res., 28, 743–746.

[ele12745-bib-0032] Kuijper, B. & Hoyle, R.B. (2015). When to rely on maternal effects and when on phenotypic plasticity? Evolution (N. Y)., 69, 950–968. 25809121 10.1111/evo.12635 PMC4975690

[ele12745-bib-0033] Lesser, K.J. , Paiusi, I.C. & Leips, J. (2006). Naturally occurring genetic variation in the age‐specific immune response of Drosophila melanogaster. Aging Cell, 5, 293–295. 16803580 10.1111/j.1474-9726.2006.00219.x

[ele12745-bib-0034] Levin, B.R. (1996). The evolution and maintenance of virulence in microparasites. Emerg. Infect. Dis., 2, 93–102. 8903208 10.3201/eid0202.960203 PMC2639826

[ele12745-bib-0035] Lindström, J. (1999). Early development and fitness in birds and mammals. Trends Ecol. Evol., 14, 343–348. 10441307 10.1016/s0169-5347(99)01639-0

[ele12745-bib-0036] Little, T.J. & Colegrave, N. (2016). Caging and uncaging genetics. PLoS Biol., 14, 1–5. 10.1371/journal.pbio.1002525 PMC4961361 27458971

[ele12745-bib-0037] Little, T.J. , O'Connor, B. , Colegrave, N. , Watt, K. & Read, A.F. (2003). Maternal transfer of strain‐specific immunity in an invertebrate. Curr. Biol., 13, 489–492. 12646131 10.1016/s0960-9822(03)00163-5

[ele12745-bib-0038] Lorenz, L.M. & Koella, J.C. (2011). Maternal environment shapes the life history and susceptibility to malaria of Anopheles gambiae mosquitoes. Malar. J., 10, 382. 22188602 10.1186/1475-2875-10-382 PMC3269443

[ele12745-bib-0039] Marshall, D.J. , Heppell, S.S. , Munch, S.B. & Warner, R.R. (2010). The relationship between maternal phenotype and offspring quality: do older mothers really produce the best offspring? Ecology, 91, 2862–2873. 21058547 10.1890/09-0156.1

[ele12745-bib-0040] Mitchell, S.E. & Read, A.F. (2005). Poor maternal environment enhances offspring disease resistance in an invertebrate. Proc. Biol. Sci., 272, 2601–2607. 16321782 10.1098/rspb.2005.3253 PMC1559984

[ele12745-bib-0041] Müller, J. (2000). Optimal vaccination patterns in age‐structured populations: endemic case. Math. Comput. Model., 31, 149–160.

[ele12745-bib-0042] Nussey, D.H. , Coulson, T. , Festa‐Bianchet, M. & Gaillard, J.‐M. (2008). Measuring senescence in wild animal populations: towards a longitudinal approach. Funct. Ecol., 22, 393–406.

[ele12745-bib-0043] Piñera, A.V. , Charles, H.M. , Dinh, T.A. & Killian, K.A. (2013). Maturation of the immune system of the male house cricket. Acheta domesticus. J. Insect Physiol., 59, 752–760. 23727197 10.1016/j.jinsphys.2013.05.008

[ele12745-bib-0044] Plaistow, S.J. , St. Clair, J.J.H. , Grant, J. & Benton, T.G. (2007). How to put all your eggs in one basket: empirical patterns of offspring provisioning throughout a mother's lifetime. Am. Nat., 170, 520–529. 17891731 10.1086/521238

[ele12745-bib-0045] Priest, N.K. , Mackowiak, B. & Promislow, D.E.L. (2002). The role of parental age effects on the evolution of aging. Evolution, 56, 927–935. 12093028 10.1111/j.0014-3820.2002.tb01405.x

[ele12745-bib-0046] Prior, N.H. , Washington, C.N. , Housley, J.M. , Hall, S.R. , Duffy, M.A. & Cáceres, C.E. (2011). Maternal effects and epidemiological traits in a planktonic host‐parasite system. Evol. Ecol. Res., 13, 401–413.

[ele12745-bib-0047] Rahman, M.M. , Roberts, H.L.S. , Sarjan, M. , Asgari, S. & Schmidt, O. (2004). Induction and transmission of Bacillus thuringiensis tolerance in the flour moth Ephestia kuehniella. Proc. Natl Acad. Sci. U. S. A., 101, 2696–2699. 14978282 10.1073/pnas.0306669101 PMC365683

[ele12745-bib-0048] Räsänen, K. & Kruuk, L.E.B. (2007). Maternal effects and evolution at ecological time‐scales. Funct. Ecol., 21, 408–421.

[ele12745-bib-0049] Read, A.F. (1994). The evolution of virulence. Trends Microbiol., 2, 73–76. 8156274 10.1016/0966-842x(94)90537-1

[ele12745-bib-0050] Rheins, L.A. & Karp, R.D. (1985). Ontogeny of the invertebrate humoral immune response: studies on various developmental states of the American cockroach. Dev. Comp. Immunol., 9, 395–406. 4043479 10.1016/0145-305x(85)90003-5

[ele12745-bib-0051] Schmid‐Hempel, P. (2011). Evolutionary Parasitology, 1st edn Oxford University Press, New York.

[ele12745-bib-0052] Stjernman, M. & Little, T.J. (2011). Genetic variation for maternal effects on parasite susceptibility. J. Evol. Biol., 24, 2357–2363. 21848987 10.1111/j.1420-9101.2011.02363.x

[ele12745-bib-0053] Tidbury, H.J. , Pedersen, A.B. & Boots, M. (2011). Within and transgenerational immune priming in an insect to a DNA virus. Proc. Biol. Sci., 278, 871–876. 20861049 10.1098/rspb.2010.1517 PMC3049047

[ele12745-bib-0054] Wilson‐Rich, N. , Dres, S.T. & Starks, P.T. (2008). The ontogeny of immunity: development of innate immune strength in the honey bee (Apis mellifera). J. Insect Physiol., 54, 1392–1399. 18761014 10.1016/j.jinsphys.2008.07.016

