# Supplementary material for: Disease spread in age structured populations with maternal age effects
Source: Ecol Lett. 2017 Mar 7;20(4):445–51. doi: 10.1111/ele.12745 (PMC6849612; doi:10.1111/ele.12745)
Supplement: Supplementary file 1 [file ELE-20-445-s001.docx]

**Appendix**

**Details of the epidemiological model**

We develop a simple age-structured model to examine the effects of age-related and maternal age related susceptibility to infection on the vulnerability of host populations to pathogen outbreaks. In the absence of the pathogen we write the dynamics of the four age classes as

|  | $\frac{dU_{Y,Y}}{dt}=r\left( U_{Y,Y}+U_{Y,O} \right)\left( 1-\frac{N_{U}}{K} \right)-\left( m+\delta\right)U_{Y,Y}$ | 5a |
| --- | --- | --- |
|  | $\frac{dU_{Y,O}}{dt}=r\left( U_{O,Y}+U_{O,O} \right)\left( 1-\frac{N_{U}}{K} \right)-\left( m+\delta\right)U_{Y,O}$ | 5b |
|  | $\frac{dU_{O,Y}}{dt}=mU_{Y,Y}-\delta U_{O,Y}$ | 5c |
|  | $\frac{dU_{O,O}}{dt}=mU_{Y,O}-\delta U_{O,O}$ | 5d |
|  | $N_{U}=U_{Y,Y}+U_{Y,O}+U_{O,Y}+U_{O,O}$ | 5e |

Solving for the equilibrium densities of each host age class we get equation 1a-1d.

At this equilibrium the total density of uninfected hosts is given by equation 2.

The population dynamics in the presence of the pathogen are given by

|  | $\frac{dU_{Y,Y}}{dt}=r\left( U_{Y,Y}+U_{Y,O} \right)\left( 1-\frac{N}{K} \right)-\left( m+\delta\right)U_{Y,Y}-\beta_{Y,Y}\left( \alpha+p\delta\right)N_{I}U_{Y,Y}$ | 6a |
| --- | --- | --- |
|  | $\frac{dU_{Y,O}}{dt}=r\left( U_{O,Y}+U_{O,O} \right)\left( 1-\frac{N}{K} \right)-\left( m+\delta\right)U_{Y,O}-\beta_{Y,O}\left( \alpha+p\delta\right)N_{I}U_{Y,O}$ | 6b |
|  | $\frac{dU_{O,Y}}{dt}=mU_{Y,Y}-\delta U_{O,Y}-\beta_{O,Y}\left( \alpha+p\delta\right)N_{I}U_{O,Y}$ | 6c |
|  | $\frac{dU_{O,O}}{dt}=mU_{Y,O}-\delta U_{O,O}-\beta_{O,O}\left( \alpha+p\delta\right)N_{I}U_{O,O}$ | 6d |
|  | $\frac{dI_{Y,Y}}{dt}=\beta_{Y,Y}\left( \alpha+p\delta\right)N_{I}U_{Y,Y}-\left( m+\alpha+\delta\right)I_{Y,Y}$ | 6e |
|  | $\frac{dI_{Y,O}}{dt}=\beta_{Y,O}\left( \alpha+p\delta\right)N_{I}U_{Y,O}-\left( m+\alpha+\delta\right)I_{Y,O}$ | 6f |
|  | $\frac{dI_{O,Y}}{dt}=\beta_{O,Y}\left( \alpha+p\delta\right)N_{I}U_{O,Y}-\left( \alpha+\delta\right)I_{O,Y}+mI_{Y,Y}$ | 6g |
|  | $\frac{dI_{O,O}}{dt}=\beta_{O,O}\left( \alpha+p\delta\right)N_{I}U_{O,O}-\left( \alpha+\delta\right)I_{O,O}+mI_{Y,O}$ | 6h |
|  | $N_{U}=U_{Y,Y}+U_{Y,O}+U_{O,Y}+U_{O,O}$ | 6i |
|  | $N_{I}=I_{Y,Y}+I_{Y,O}+I_{O,Y}+I_{O,O}$ | 6j |
|  | $N=N_{U}+N_{I}$ | 6k |

Note that as *P. ramosa* transmits at host death, transmission between infected and uninfected hosts is weighted by $\left( \alpha+p\delta\right)$, where $\alpha$ is the pathogen induced death rate, which is assumed to always facilitate transmission, and *p* is the proportion of non-pathogen induced death that also facilitates transmission. From these dynamics we can evaluate the number of expected secondary infections caused by a rare infection as

|  | $R_{0}=\frac{\left( \alpha+p\delta\right)\left( \beta_{Y,Y}U_{Y,Y}+\beta_{Y,O}U_{Y,O}+\beta_{O,Y}U_{O,Y}+\beta_{O,O}U_{O,O} \right)}{\alpha+\delta}$ | 7 |
| --- | --- | --- |

In order to evaluate the effects of age-class specific susceptibilities on the *R*_0_ we define the effects of age-related and maternal age related reduction in susceptibility as

|  | $\beta_{Y,Y}=\beta$ | 8a |
| --- | --- | --- |
|  | $\beta_{O,Y}=\beta\left( 1-A \right)$ | 8b |
|  | $\beta_{Y,O}=\beta\left( 1-M \right)$ | 8c |
|  | $\beta_{O,O}=\beta\left( 1-A \right)\left( 1-M \right)$ | 8d |

where $0\leq A\leq1$ and $0\leq M\leq1$ are the proportional reductions in susceptibility owing to age and maternal age respectively, which here we assume to interact multiplicatively (though see later for additive effects). Inserting equations 1a-1d and 8a-8d into equation 7 we get the expression given in equation 3.

To evaluate the effect of non-pathogen induced mortality, *δ*, on the pathogen *R*_0_ we first consider the derivative of *R*_0_ with respect to *δ* as *δ* approaches infinity, which gives

|  | $\lim_{\delta\to\infty} \frac{dR_{0}}{d\delta}=-\frac{\beta pK}{r}$ | 9 |
| --- | --- | --- |

which is strictly negative, meaning that at very high values of *δ* the pathogen *R*­_0_ must decline as the host population approaches zero. Similarly, if we consider the behavior of this derivative as *δ* approaches zero we get

|  | $\lim_{\delta\to0} \frac{dR_{0}}{d\delta}=\frac{\beta K}{\alpha mr}\left( \alpha\left( r\left( A+M-2AM \right)-m\left( 1-A \right)\left( 1-M \right) \right)-mr\left( 1-A \right)\left( 1-M \right)\left( 1-p \right) \right)$ | 10 |
| --- | --- | --- |

which is positive whenever inequality 4 is satisfied. This inequality can never be satisfied for *A* = 0 and *M* = 0, meaning that in the absence of age and maternal age based reductions in susceptibility the pathogen *R*_0_ always decreases with increasing mortality. However, this inequality can be satisfied for sufficiently high values of *A* and *M*, meaning that pathogen *R*_0_ can initially increase with non-pathogen induced mortality.

**Additive age and maternal age effects.**

In equation 8 we assume that age and maternal age interact multiplicatively. Here we show that our qualitative results also hold for additive effects. In this case the susceptibilities for each age class are

|  | $\beta_{Y,Y}=\beta$ | 11a |
| --- | --- | --- |
|  | $\beta_{O,Y}=\beta\left( 1-A \right)$ | 11b |
|  | $\beta_{Y,O}=\beta\left( 1-M \right)$ | 11c |
|  | $\beta_{O,O}=\beta\left( 1-A-M \right)$ | 11d |

Now the number of expected secondary infections caused by a rare infection is

|  | $R_{0}=\frac{\gamma\beta\left( r-\delta\right)\left( \alpha+\delta p \right)\left( \delta+m\left( 1-M-A \right) \right)K}{\lambda r\left( \alpha+\delta\right)\left( \delta+m \right)^{2}}$ | 12 |
| --- | --- | --- |

We can again calculate the derivative of *R*_0_ with respect to *δ* as *δ* approaches infinity, which gives

|  | $\lim_{\delta\to\infty} \frac{dR_{0}}{d\delta}=-\frac{\beta pK}{r}$ | 13 |
| --- | --- | --- |

which is again strictly negative, meaning that at very high values of *δ* the pathogen *R*­_0_ must decline as the host population approaches zero. Similarly, if we consider the behavior of this derivative as *δ* approaches zero we get

|  | $\lim_{\delta\to0} \frac{dR_{0}}{d\delta}=\frac{\gamma\beta K}{\alpha mr}\left( \alpha\left( r\left( A+M \right)-m\left( 1-A-M \right) \right)-mr\left( 1-A-M \right)\left( 1-p \right) \right)$ | 14 |
| --- | --- | --- |

which is positive whenever

|  | $m<\frac{\alpha r\left( A+M \right)}{\left( 1-A-M \right)\left( \alpha+r\left( 1-p \right) \right)}$ | 15 |
| --- | --- | --- |

Again this inequality can never be satisfied for *A* = 0 and *M* = 0, meaning that in the absence of age and maternal age based reductions in susceptibility the pathogen *R*_0_ always decreases with increasing mortality. However, this inequality can be satisfied for sufficiently high values of *A* and *M*, meaning that pathogen *R*_0_ can initially increase with non-pathogen induced mortality, matching our qualitative results with multiplicative effects.

**Including environmental transmission stages.**

In our main model we simplify the true epidemiological dynamics of *P. ramosa* by assuming that transmission occurs directly between hosts, while in reality an environmental transmission stage also occurs (Ebert *et al.* 2016). Here we modify our model to include this environmental transmission stage. Re-writing equation 6 to include environmental transmission gives

|  | $\frac{dU_{Y,Y}}{dt}=r\left( U_{Y,Y}+U_{Y,O} \right)\left( 1-\frac{N}{K} \right)-\left( m+\delta\right)U_{Y,Y}-\beta_{Y,Y}EU_{Y,Y}$ | 16a |
| --- | --- | --- |
|  | $\frac{dU_{Y,O}}{dt}=r\left( U_{O,Y}+U_{O,O} \right)\left( 1-\frac{N}{K} \right)-\left( m+\delta\right)U_{Y,O}-\beta_{Y,O}EU_{Y,O}$ | 16b |
|  | $\frac{dU_{O,Y}}{dt}=mU_{Y,Y}-\delta U_{O,Y}-\beta_{O,Y}EU_{O,Y}$ | 16c |
|  | $\frac{dU_{O,O}}{dt}=mU_{Y,O}-\delta U_{O,O}-\beta_{O,O}EU_{O,O}$ | 16d |
|  | $\frac{dI_{Y,Y}}{dt}=\beta_{Y,Y}EU_{Y,Y}-\left( m+\alpha+\delta\right)I_{Y,Y}$ | 16e |
|  | $\frac{dI_{Y,O}}{dt}=\beta_{Y,O}EU_{Y,O}-\left( m+\alpha+\delta\right)I_{Y,O}$ | 16f |
|  | $\frac{dI_{O,Y}}{dt}=\beta_{O,Y}EU_{O,Y}-\left( \alpha+\delta\right)I_{O,Y}+mI_{Y,Y}$ | 16g |
|  | $\frac{dI_{O,O}}{dt}=\beta_{O,O}EU_{O,O}-\left( \alpha+\delta\right)I_{O,O}+mI_{Y,O}$ | 16h |
|  | $\frac{dE}{dt}=\gamma\left( \alpha+p\delta\right)N_{I}-E\left( \beta_{Y,Y}U_{Y,Y}+\beta_{Y,O}U_{Y,O}+\beta_{O,Y}U_{O,Y}+\beta_{O,O}U_{O,O}+\lambda\right)$ | 16i |
|  | $N_{U}=U_{Y,Y}+U_{Y,O}+U_{O,Y}+U_{O,O}$ | 16j |
|  | $N_{I}=I_{Y,Y}+I_{Y,O}+I_{O,Y}+I_{O,O}$ | 16k |
|  | $N=N_{U}+N_{I}$ | 16l |

where *E* is the density of the environmental spores of the pathogen, *γ* is the number of pathogen spores released upon host death, and *λ* is the death rate of the environmental spores. We can then calculate the pathogen *R*_0_ as

|  | $R_{0}=\frac{\gamma\beta\left( r-\delta\right)\left( \alpha+\delta p \right)\left( \delta+m\left( 1-M \right) \right)\left( \delta+m\left( 1-A \right) \right)K}{\lambda r\left( \alpha+\delta\right)\left( \delta+m \right)^{2}}$ | 17 |
| --- | --- | --- |

We can again calculate the derivative of *R*_0_ with respect to *δ* as *δ* approaches infinity, which gives

|  | $\lim_{\delta\to\infty} \frac{dR_{0}}{d\delta}=-\frac{\gamma\beta pK}{\lambda r}$ | 18 |
| --- | --- | --- |

which is again strictly negative, meaning that at very high values of *δ* the pathogen *R*­_0_ must decline as the host population approaches zero. Similarly, if we consider the behavior of this derivative as *δ* approaches zero we get

|  | $\lim_{\delta\to0} \frac{dR_{0}}{d\delta}=\frac{\gamma\beta K}{\lambda\alpha mr}\left( \alpha\left( r\left( A+M-2AM \right)-m\left( 1-A \right)\left( 1-M \right) \right)-mr\left( 1-A \right)\left( 1-M \right)\left( 1-p \right) \right)$ | 19 |
| --- | --- | --- |

which is again positive whenever inequality 4 is satisfied. Thus under environmental transmission our qualitative results hold.
